# Supplementary material for: Assessing Polysaccharides/Aloe Vera–Based Hydrogels for Tumor Spheroid Formation
Source: Gels. 2023 Jan 7;9(1):51. doi: 10.3390/gels9010051 (PMC9858450; doi:10.3390/gels9010051)
Supplement: Supplementary file 1 [file gels-09-00051-s001.zip › gels-2122275-supplementary.pdf]

Supplementary Materials

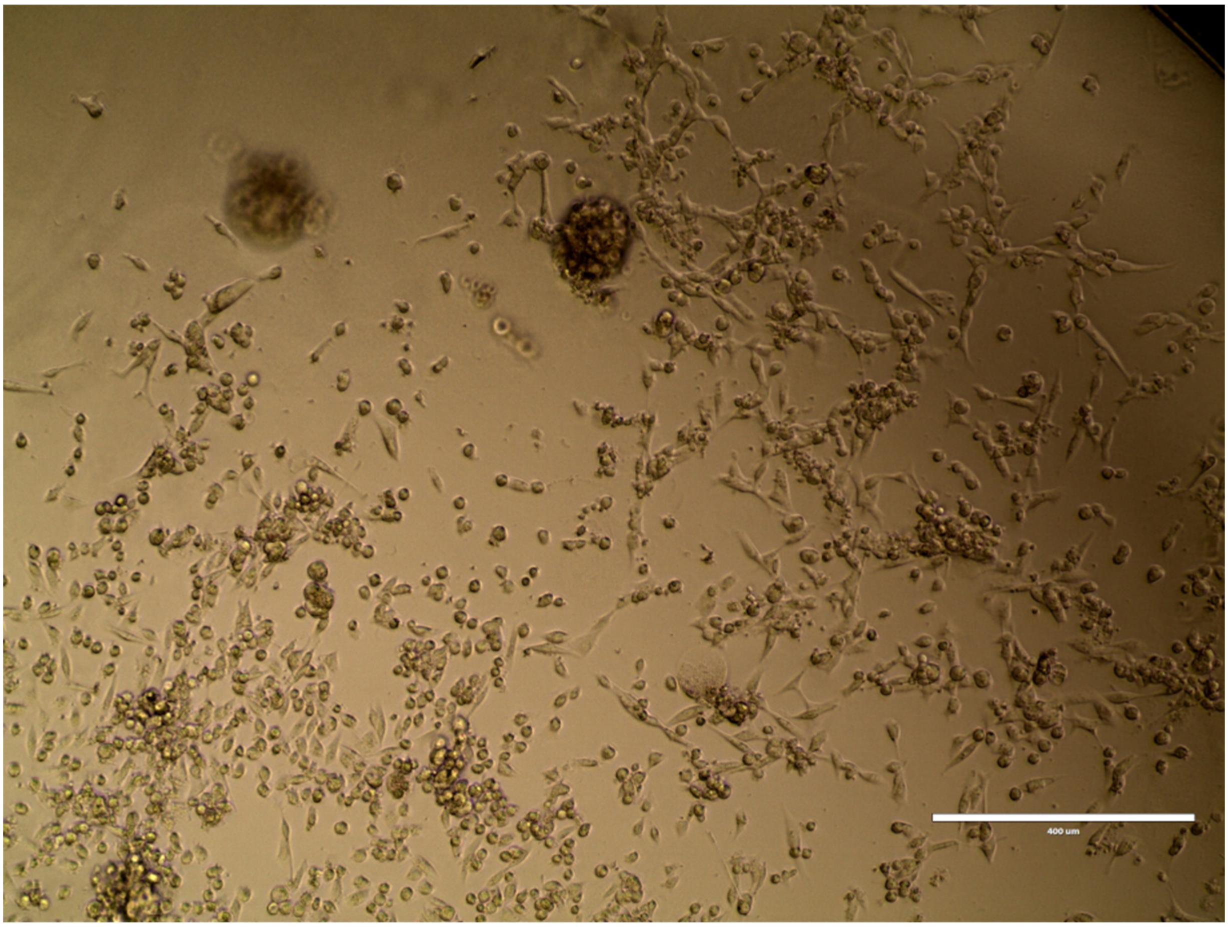

Figure S1. Image of mixed 2D and 3D cell culture of MDA-MB-231.

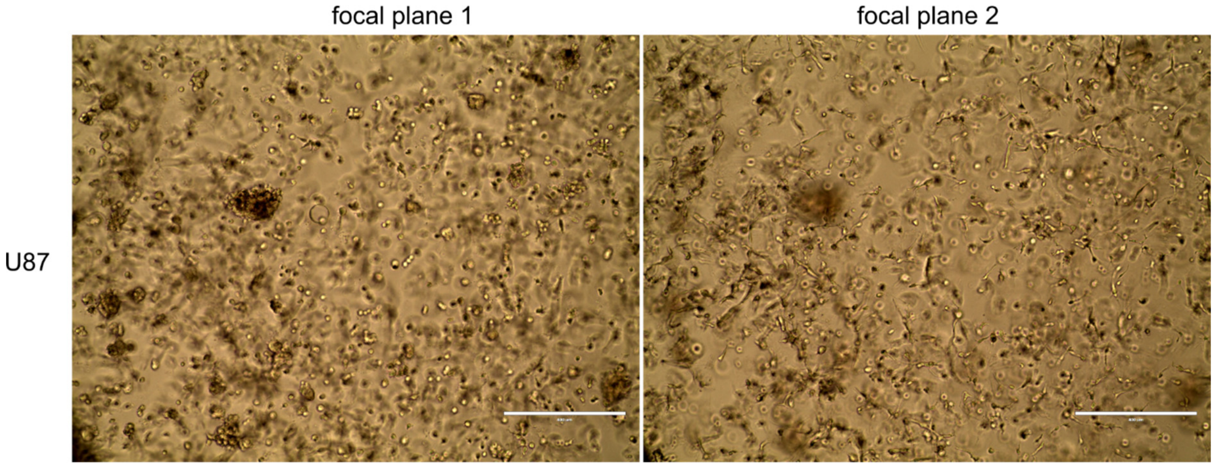

Figure S2. Images of mixed 2D and 3D cell culture of U87MG.

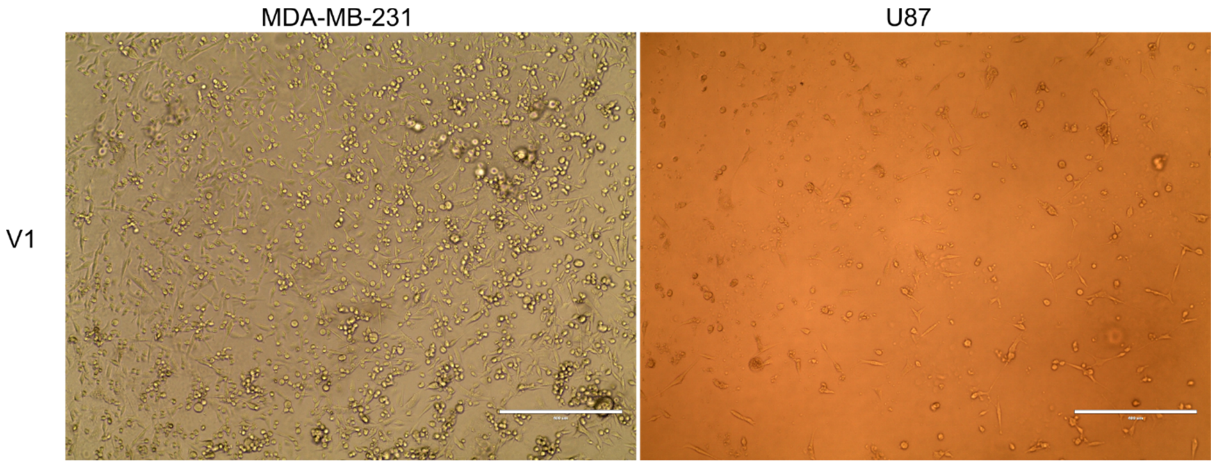

Figure S3. Image of MDA-MB-231 and U87MG cell culture developed in V1 hydrogel.

Supplementary R1 – Rheological characterisation

<u>Shear stress vs. shear rate</u>

The shear stress against shear rate rheograms for the studied hydrogels displayed in Figure 1 clearly show an increasing trend of the shear stress with increasing shear rate, which confirms the shear thinning non-Newtonian behavior of the hydrogels.

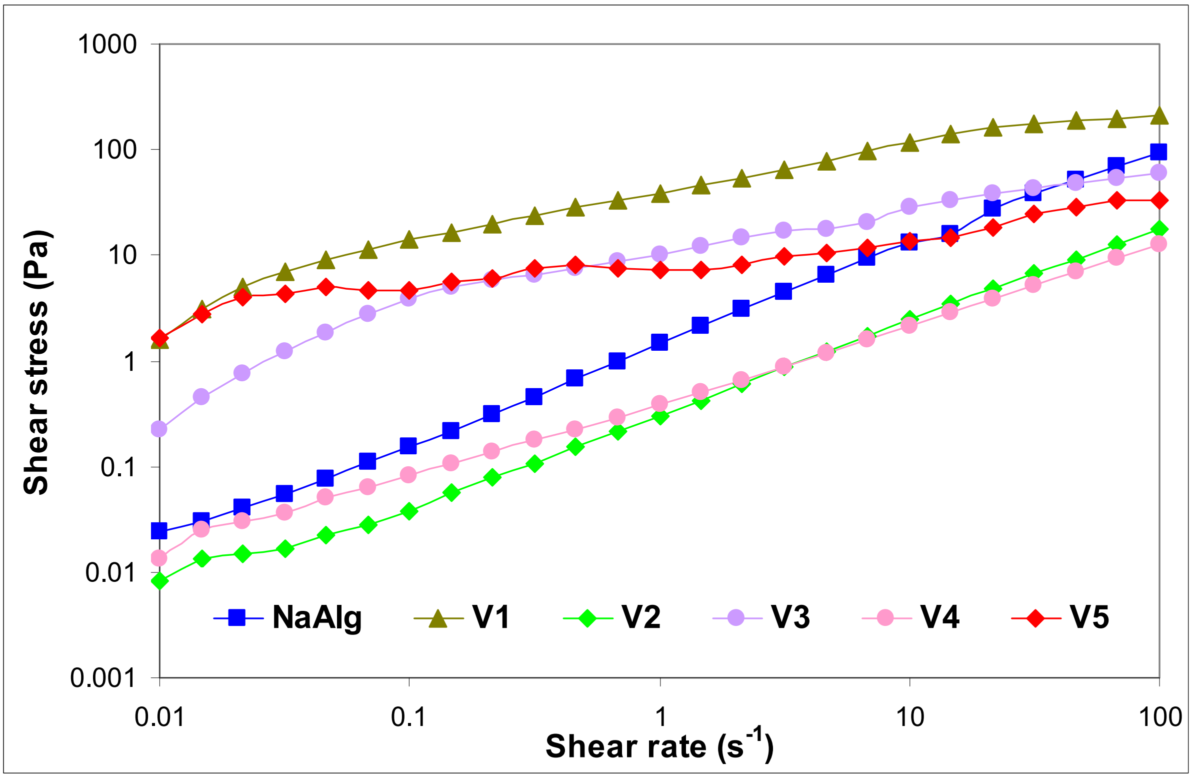

Figure 1. Rheograms (shear stress vs. shear rate) recorded for evaluated alginate-based hydrogels at different pHs and 37 °C

Shear stress versus shear rate rheograms recorded for the studied hydrogels (Figure 1) generally follow a power law. The flow behavior index (n) values were evaluated from these rheogram using the Ostwald–de Waele model [53, 67] shown in Equation (1):

$$\tau = K\dot{\gamma}^{n} \quad (1)$$

where τ is the shear-stress (Pa), $\dot{\gamma}$ is the shear-rate (s^{-1}), parameter K represents the flow consistency index, a measure of average viscosity of the fluid, and the power law index n refers to the flow behavior index, a measure of its deviation from Newtonian behavior [54]. The mathematical relationship of Ostwald–de Waele model approximately describes the behavior of a real non-Newtonian fluid.

The results presented in Table 1 show the flow behaviour index (n) has values below unity, corresponding to pseudoplastic (or shear-thinning) fluids. The obtained hydrogels prepared at acidic pH (pH 5) display higher average viscosity (K) and is also significantly enhanced shear thinning (lower n values), while the sample prepared at basic pH (pH 12) have lower flow consistency index K values, but higher flow behavior index n.

Table 1. Power law parameters and regression coefficient (R^2) evaluated from experimental rheograms

pH	Samples	K	n	R^2
5	NaAlg (2%)	1.4388	0.9323	0.9990
	V1	34.594	0.4855	0.9676
	V3	8.1559	0.5257	0.9351
	V5	8.5289	0.2626	0.9361
12	V2	0.3283	0.8515	0.9971
	V4	0.4198	0.7165	0.9986

Supplementary R2 – Rheological characterisation

Frequency sweep tests

The viscoelastic characteristics evaluated from dynamic moduli storage modulus (G′) and loss modulus (G″), as well as the complex viscosity function of oscillatory frequency outcome the results of the flow behavior tests. The pH has a strong influence on the viscoelastic properties of the evaluated hydrogels (Figure 1), the lower (acidic) the pH, the higher the strength of the resulting developed hydrogels.

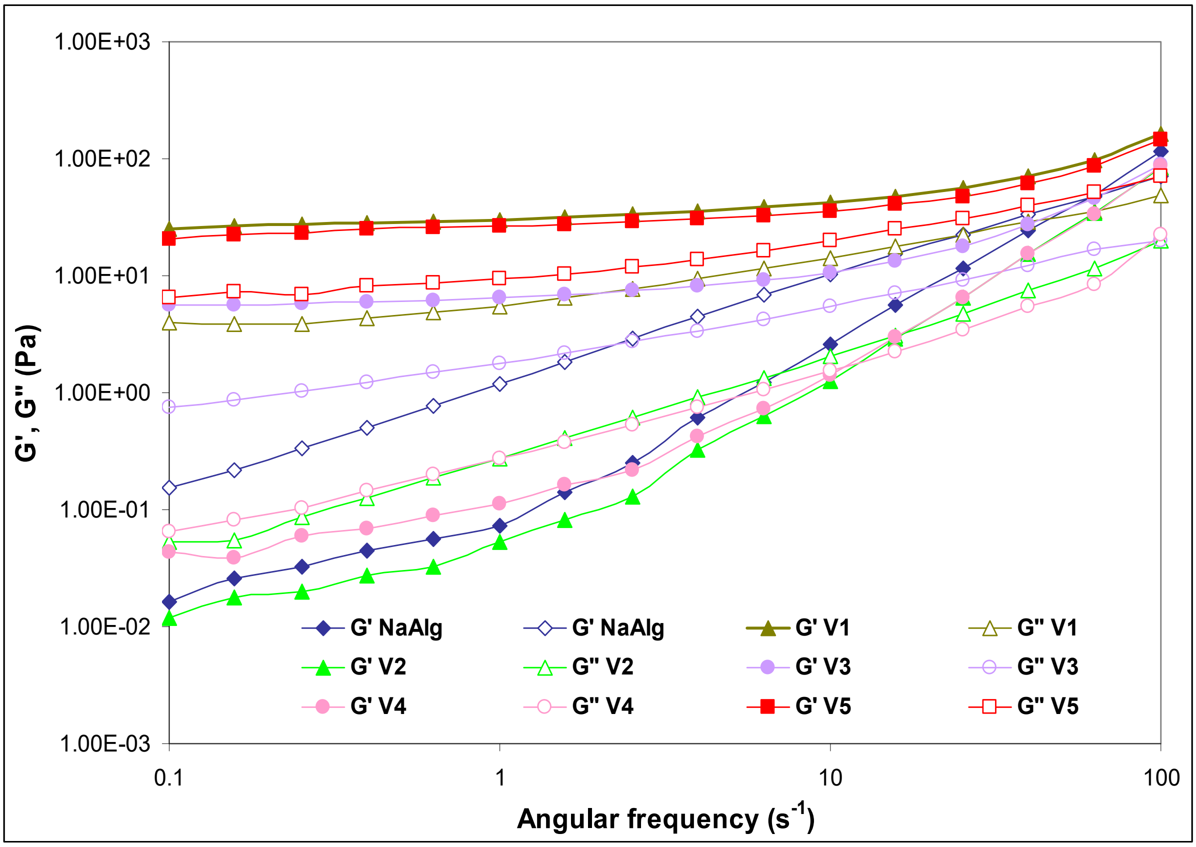
1.00E+03
1.00E+02
1.00E+01
1.00E+00
1.00E-01
1.00E-02
1.00E-03
G', G" (Pa)
0.1
1
10
100
Angular frequency (s-1)
G' NaAlg
G' NaAlg
G' V1
G" V1
G' V2
G" V2
G' V3
G" V3
G' V4
G" V4
G' V5
G" V5

(a)

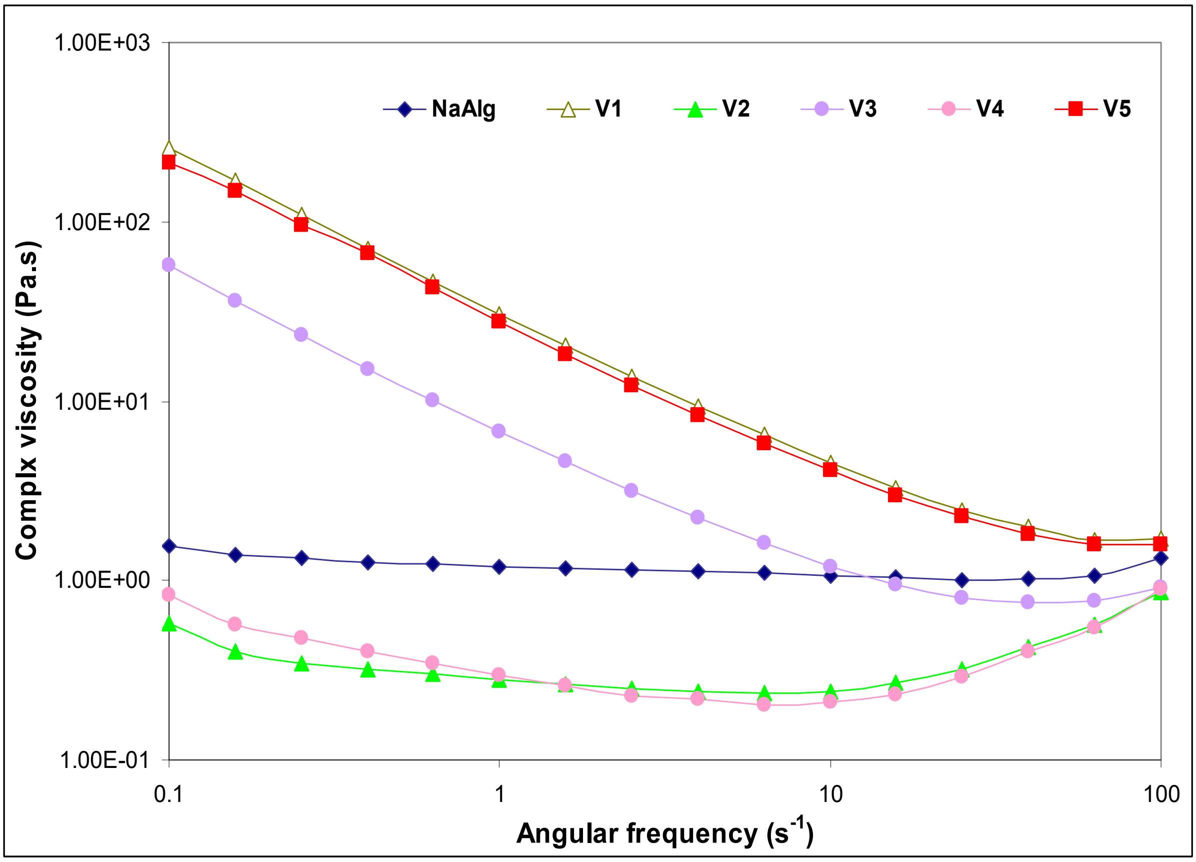

(b)

Figure 1. Dependence of (a) G′ and G″ dynamic moduli on the angular frequency and (b) complex viscosity for the alginate-based hydrogels at different pHs and 37°C

The incorporation of Aloe vera or chitosan in the alginate formulation obtained at pH 5 increased over three times the storage modulus and almost twice the loss modulus at low frequencies, resulting in hydrogels more resistant to deformation with a gel-like behavior (G′ > G″). Other studies also reported development of stiffer materials by incorporation of chitosan within alginate in different concentrations and obtaining methods [68, 69].

The complex viscosity of NaAlg sample is independent of the deformation frequency, while the hydrogels with basic pH flow easier under oscillation.
